# Segmented paths, shared beliefs? Employment histories and welfare preferences in Chile

**DOI:** 10.3389/fsoc.2026.1735350

**Published:** 2026-04-20

**Authors:** Andrés Biehl, Ignacio Cabib, Andrés González Ide

**Affiliations:** 1 Instituto de Sociología, Pontificia Universidad Católica de Chile, Santiago, Chile; 2 Escuela de Salud Pública, Pontificia Universidad Católica de Chile, Santiago, Chile; 3 RTG DYNAMICS, Humboldt-Universität zu Berlin, Berlin, Germany

**Keywords:** Chile, fiscal preferences, informality, labor market, Latin America, life course, policy preferences, sequence analysis

## Abstract

**Introduction:**

In Latin America, employment status consistently fails to predict redistributive attitudes. This is a puzzle given that labor market informality shapes access to social protection. Yet most studies rely on cross-sectional data, overlooking how cumulative work trajectories across the life course structure welfare preferences. This article examines how formal and informal employment histories shape current preferences for redistribution and taxation among Chileans born between 1938 and 1963.

**Methods:**

Using a specially designed survey with a life-history calendar administered to 792 older Chileans, we reconstruct individual employment trajectories between ages 30 and 60. Sequence analysis and hierarchical clustering identify typical trajectory types, which are then related to preferences for redistribution and taxation through logistic regression models.

**Results:**

We identify trajectories ranging from persistently formal to erratic to inactive. Individuals with erratic or inactive trajectories are significantly more likely to favor targeted benefits over universalism and less likely to hold fiscal citizenship attitudes toward broad-based domestic taxation.

**Discussion:**

These findings challenge two sets of expectations. First, they qualify the cross-sectional evidence suggesting that formal and informal workers in Latin America hold similar preferences. Second, and more surprisingly, they run counter to conventional welfare state theory, which predicts that workers with precarious or interrupted employment histories would push for universal benefits as a form of social insurance. Instead, we find the opposite: cumulative employment instability appears to breed support for targeted transfers and weaker fiscal citizenship. By adopting a life-course perspective, we show that trajectory may be a key driver of divergent welfare preferences in the region.

## 1 Introduction

In this article, we explore how formal and informal work trajectories shape the current policy preferences of Chileans born between 1938 and 1963. In doing so, we address two interrelated issues: (1) a “Latin American” empirical puzzle regarding employment and redistributive preferences, and (2) a neglected research gap concerning preferences for tax policy. Taken together, our paper contributes to both academic and policy debates on the rapid recent expansion of non-contributory social protection in the region (Barrientos, 2013, 2023; Böger and Leisering, 2020; Melguizo et al., 2015).

First, the empirical puzzle emerges from the comparative analysis of policy preferences in Latin America, North America, and Europe, and, perhaps, from carrying the assumptions of more affluent labor markets into Latin American realities (Biehl et al., 2024). Research on more generous welfare states and richer economies has documented clear links between labor market position and attitudes to political and redistributive preferences (Chan and Goldthorpe, 2007; Emmenegger et al., 2012; Fernandez-Albertos and Manzano, 2014; Gingrich and Ansell, 2012; Kulin and Svallfors, 2013; Tomlinson and Walker, 2012). Following an insider and outsider framework, formal and informal workers in these contexts are often seen as occupying distinct, stable positions that rationally inform their policy demands (Rueda, 2005, 2006). Typically, formal workers are already covered by social insurance to which they contribute and would resist the expansion of tax-funded, non-contributory programs. They would favor, instead, stable employment and further regulation. Informal workers, by contrast, are expected to demand broader, tax-based, social protection and unemployment benefits of a more universal character.

Yet, despite segmentation and entrenched inequalities, Latin American workers fail to display such rigidly opposing policy preferences (Berens, 2015a). As Baker and Velasco-Guachalla (2018) argue, individual employment histories often combine periods of informality and formality while households often articulate coresidential strategies based on formal and informal work between family members (see also Araos and Siles, 2021; Barrientos and Lloyd-Sherlock, 2003; Madero-Cabib and Biehl, 2021; Palacios, 2011; Tokman, 2012). As a result, a clear-cut informal/formal distinction would hold limited sway over their individual preferences. Given their distinct labor markets, why would Latin Americans exhibit the same sharp policy cleavages of their European and North American counterparts? In this context, assuming the same clear distinctions of more affluent settings would, at best, inform a quasi-empirical puzzle for a region where informality and formality seem less as discrete categories than points along a continuum, and thus fail to shape distinct life course paths.

Crucially, most of existing studies rely on cross-sectional data, limiting our ability to observe how employment trajectories evolve over time and influence current policy preferences. Baker and Velasco-Guachalla’s (2018) “non-results” touch on this problem. Using the Argentine Panel Election Study of 2015, they test this possibility comparing two waves, between June and August, which yield significant differences in preferences, with persistent informal workers welcoming non-contributory programs. However, they caution against making too much of these findings because of the limited sample size and the short time frame between waves. And yet, even if labor market segmentation is fluid rather than fixed, we could still expect variation within different trajectories. We just do not know them. In other words, the complexity of work histories could still hide significant differences in policy preferences between those that are persistently formal, persistently informal, and the grey area of more erratic paths combining different statuses at different career stages. To address this issue, we employ a life course perspective and exploit a specially designed data set combining retrospective work information and current social policy attitudes.

Second, as well as looking at spending preferences, we address tax policy which is beginning to draw increasing interest among scholars (Atria et al., 2019; Berens and von Schiller, 2017; Torgler, 2005). If differences on spending options are shaped by workers’ employment status, we could expect that these differences arise not only from expected benefits but also from their perceived costs. In this vein, we explore whether employment trajectories affect current attitudes toward taxation. This is particularly important for current debates on funding the expansion of non-contributory benefits across the region given, among others, the rise of public debt and limits to funding mechanisms (Barrientos, 2013; Böger and Leisering, 2020; Larrañaga, 2025; Melguizo et al., 2015).

In what follows, we elaborate on the theoretical background, research gaps, and case selection, then outline data and analytical strategy, and finally we discuss our results and limitations.

## 2 Background: spending, taxation, and older Chileans

### 2.1 Spending and redistributive preferences

Labor market segmentation between formal and informal workers creates distinct incentives, a structure of costs and benefits, that should translate into policy preferences (Alt and Iversen, 2017; Emmenegger et al., 2012; Rueda, 2006). Research in wealthier nations and more comprehensive welfare states, documents significant differences. Formal workers, covered by contracts and social security, risk losing stability which is why they would support regulation fostering stable employment that, in turn, funds their future pensions and health insurance (Alt and Iversen, 2017; Häusermann and Schwander, 2012; Tomlinson and Walker, 2012). In contrast, informal workers, usually on short-term contracts or not covered by social security, often demand unemployment benefits and tax-funded social security of a more universal kind (Fernandez-Albertos and Manzano, 2014; Rueda, 2006; Tomlinson and Walker, 2012). The logic being, that insiders, who contribute to welfare through payroll taxes, would resist non-contributory benefits, while outsiders would support them, thus shaping a clear collective action problem as formal workers might see informal workers as potential free riders.

As Häusermann and Schwander (2012) argue, welfare states often compensate for labor market segmentation but simultaneously reinforce occupational inequalities by allocating different forms of redistribution to formal and informal workforces. Facing different risks, costs, and benefits, informal workers tend to support welfare expansion to offset the limited protection they receive, while formal workers appear more ambivalent about supporting spending that ultimately relies on their social contributions or income taxes. Informal workers are expected to favor short-term compensatory policies such as unemployment benefits, or use informality as a safety net, while formal workers prioritize long-term goals such as pensions and health coverage (Burgoon and Dekker, 2010; Loayza and Rigolini, 2011).

However, despite entrenched labor market segmentation in Latin America, formal and informal workers, though exposed to different opportunities and vulnerabilities, exhibit similar redistributive and political preferences. In Latin America, then, this question is all the more relevant given the persistence of informality, precarity, and labor market segmentation. Berens (2015a) tests for whether these groups favor different redistributive policies. Yet, while she shows that these groups indeed face differing incentives and risks, both groups express similar support for expanding social, non-contributory, benefits widely across the population. This suggests that, despite horizontal inequalities in the labor market, workers’ preferences in the region may not be shaped by segmentation. In later research, Berens documents that outsiders are indeed a more complex category: in Mexico, for instance, vulnerable populations could sometimes resist welfare expansion and, faced with a large informal sector, formal workers across Latin America may opt for private provision of health and pensions (Altamirano et al., 2022; Berens, 2015b). Even more puzzling, informal workers show support for restrictive labor laws, particularly a higher minimum wage, that increases the cost of formalization (Ronconi et al., 2023; but see Berens and Kemmerling, 2019). And yet, the poor and vulnerable to economic shocks seem to demand more redistribution (Busso et al., 2025).

These results are puzzling for two reasons. First, the historical development of Latin American welfare states along Bismarckian lines has reinforced segmentation and stratification by formality and occupation (Barrientos, 2025; Haggard and Kaufman, 2008; Huber and Bogliaccini, 2010). Informal and family-based care systems complement or offer a safety net to precarious labor markets (Holland, 2017; Pribble, 2011; Centeno and Portes, 2006).

Second, Latin American countries have witnessed a marked expansion of non-contributory pensions (Barrientos, 2023; Brearley, 2016; Mesa-Lago, 2021). The reasons behind this shift remain contested: some accounts emphasize the role of political coalitions and left-wing governments, while others point to the influence of technocratic experts and conservative economists (Blofield, 2019; Carnes and Mares, 2014; Dorlach, 2021; Martínez Franzoni and Sánchez-Ancochea, 2016). Yet the trajectories toward quasi-universal, tax-funded welfare have varied across the region. In some cases, policies were introduced in line with prevailing national preferences (Carnes and Mares, 2013); in others, most notably Mexico, they were first tried and gained traction at the subnational level, where a demonstrative effect paved the way for subsequent national adoption (Medrano, 2022; Medrano Buenrostro and Velázquez Leyer, 2023). Non-contributory programs have generally been welcomed, saw heightened demand after COVID, and participation in them further encourages their appeal (Busso et al., 2025; Inzunza and Madeira, 2023; Schwarz and Stampini, 2025).

We follow Baker and Velasco-Guachalla (2018) who suggest that the absence of differences in redistributive preferences between insiders and outsiders may be due to the fluidity of employment trajectories. Rather than experiencing stable formal or informal careers, many workers alternate between statuses, leading to similar lived experiences and, probably, policy preferences (Cabib et al., 2026a; OECD, 2024; Ward, 2019). However, we hypothesize that individuals with predominantly formal employment may develop distinct preferences. Most research in Latin America relies on cross-sectional data. Given that workers in the region frequently alternate between formal and informal employment and follow erratic career paths (for Chile, arguably one of the most formal settings, see: Madero-Cabib et al., 2019 or Madero-Cabib and Cabello-Hutt, 2022, and even large turnover within formal workers, see Albagli et al., 2023) current employment status may not adequately reflect their long-term labor market experiences. To address this, we reconstruct employment trajectories longitudinally to assess whether sustained exposure to formality or informality influences policy preferences.

Specifically, we test two hypotheses regarding formal employment trajectories on spending preferences and redistribution. First, individuals with predominantly formal trajectories will prefer more targeted rather than universal social expenditures, reflecting their continuous contributions to social security and a residual preoccupation with poverty (H1). Second, they will favor merit-based over egalitarian income distribution, i.e., tolerate more inequality, given their support for contributory social insurance (H2). Consistently informal workers and those following more erratic paths, accordingly, would prefer means-tested broad social policies funded by general taxation and more egalitarian outcomes. Implicitly, this would mean that the growth of support for non-contributory spending both among formal and informal workers results from the deficiencies of contributory systems and/or low trust in them (Arnold et al., 2024; Berens and von Schiller, 2017; Espacio Público, OIT, PNUD, & IPSOS et al., 2024).

Naturally, both hypotheses are debatable. H1 follows on the footsteps of more affluent contexts. Against H2, informality in Latin America has often been connected to capitalist and individualist values that may push for preferences against a bigger role of the state (de Soto, 1986) and, indeed, vulnerable populations not always push for increased welfare (Altamirano et al., 2022). And yet, the growth of vulnerable middle classes may have shaped a distinct demand for more universal and egalitarian social spending in recent times that has found political voice through new non-contributory policies (Barrientos, 2025; Borges, 2022; Busso et al., 2025).

Connecting experiences of formality and informality with debates on the individualistic ethos (Araujo and Martuccelli, 2020) of many Latin American workers, we test two additional hypotheses. Since the 1990s, the expansion of education and the promise of merit-based social mobility have played a central role in the region’s modernization process. Previous research has shown that individualistic attitudes are usually offered to legitimize economic outcomes (Castillo et al., 2019; Frei et al., 2020). Targeting aid and middle class opting out of public services may foster greater acceptance of inequality and increase demand for residual, targeted redistribution (Berens, 2015b; Campos-Vazquez et al., 2022; De La O et al., 2025; Torche, 2014). Building on this, we expect that individuals who experienced upward educational mobility (relative to their parents) will be more likely to endorse individualistic views, specifically: support for targeted social spending (H3a) and greater acceptance of inequality (H3b).

### 2.2 Taxation preferences

Latin American preferences for taxation policies are gaining scholarly interest (Berens and von Schiller, 2017; Castillo and Olivos, 2014; Torgler, 2005). Historically, the region has been characterized by the absence of fiscal citizenship, which was central for the expansion of welfare states and social spending in Europe and North America (Biehl and Labarca, 2018; Centeno, 2002; Mares and Queralt, 2015; Scheve and Stasavage, 2016; Timmons, 2005). The weak development of fiscal citizenship and domestic income taxation has been linked to reliance on commodities and natural resources as the main drivers of state revenue, as well as to the relative invisibility of mass domestic taxation instruments such as the value-added tax (VAT) (Biehl et al., 2025; Atria et al., 2019; Biehl and Labarca, 2018). In this context, the question of which taxation tools should be prioritized becomes especially important at a time when tax-funded and debt-driven expenditures are expanding (Melguizo et al., 2015; Mesa-Lago, 2021; Larrañaga, 2025).

Unsurprisingly, research in the region has provided a complex picture of fiscal preferences. While support for redistribution seems widespread, focalization of expenditures, low trust, perceived inefficiency, and crime often undermine compliance and limit efforts to increase revenue (Atria, 2019; Berens and von Schiller, 2017; Bergman, 2009; Busso et al., 2025; Castañeda-Rodríguez, 2025). Higher tax levels remain politically unpopular though progressivity and taxation of commodities receive backing (Ardanaz et al., 2022; Ardanaz and Dvoskin, 2008; García-Sánchez et al., 2022). Support for tax-funded redistribution is undermined when seen as to be narrowly “pro-poor” (Berens and Bastiaens, 2024) or could face backlash when targeted to stigmatized groups (Joseph et al., 2025). Perceived inequality restricts demands for fiscal citizenship or mass domestic taxation (Heilbrun, 2025; but see García-Sánchez et al., 2022). Alternatively, broad-based expenditures may lead in future to legitimize higher taxation (Barrientos, 2023, 2025).

Following employment logics, if redistributive preferences are shaped by labor market segmentation, they are typically assumed to mirror the costs borne by workers in funding social security. Formal workers, already covered by contributory systems, are expected to resist higher taxation, while informal workers, facing income insecurity, are presumed to favor non-contributory or tax-funded protection.

Building on this, we hypothesize that those following formal employment trajectories will support mass taxation (H4). Their support would be indicative of what the literature terms fiscal citizenship as taxpayers would expect obligations to be widely shared. Formal workers, having developed a closer relationship with the tax system, may prefer broad-based taxation over class-based taxation (i.e., taxing only the wealthy). Given their stable engagement with tax institutions and higher awareness of the fiscal costs of state spending, we also expect that formal workers will favor domestic taxation over commodity-based taxation as the primary means of funding the state (H5).

### 2.3 Older Chileans today

To address the conceptual and empirical gaps on how labor market dynamics shape policy preferences, we focus on older Chileans. Specifically, we analyze the employment trajectories of the 1938–1963 cohort that lives in Santiago. This cohort entered the labor market during the neoliberal restructuring of the Pinochet era and began retiring in the 2010s, just as non-contributory pensions were introduced and expanded to offset the inequalities and limitations of individual pension accounts (Cabib et al., 2024; Mesa-Lago and Bertranou, 2016).

Employing data of the Longitudinal Protection Social Survey (ELPS) Madero-Cabib et al. (2019) document that older Chileans followed a complex set of work trajectories. Using sequence analysis, they identified seven work trajectories. The largest group (44%) followed a conventional path of continuous, formal, full-time wage employment, reflecting the dominant “standard” career model particularly among men. A sizable share (31.4%) remained largely out of the labor force, moving between inactivity, unemployment, or job search. Another 11.2% pursued full-time self-employment throughout their lives, while smaller groups combined wage work or self-employment with irregular or part-time patterns: wage earners outside stable contracts (5.3%), full-time self-employed workers (3.8%), part-time self-employed (2.5%), and part-time wage earners (1.9%). Together, these trajectories reveal both the persistence of conventional careers and the prevalence of more erratic employment paths across this cohort.

Although this generation entered the labor force just as private pension contributions were introduced, they now rely primarily on non-contributory pensions and public health care for income and health security (Larrañaga, 2025). Studying this cohort then allows both the reconstruction of full employment trajectories and a focus on the group most affected by recent pension reforms. Entering the workforce in the late 1970s and early 1980s, when Chile’s economic conditions resembled those of much of the region, they experienced the shift from a contributory, state-based pension model to a system of defined contributions into individual accounts run by private fund administrators (AFPs). Their working lives unfolded during the economic growth of the 1990s, yet they began to retire in the 2010s under conditions of slower growth and greater uncertainty. This sequence of structural changes makes them an interesting case for examining how long-term employment trajectories shape contemporary preferences for welfare expansion and taxation. Their current redistributive preferences are thus likely shaped by employment histories, contribution densities, and post-retirement work, alongside conventional predictors such as education and political orientation.

## 3 Data and analytical strategy

To explore these questions, we conducted a full-probability survey which includes 792 individuals residing in Santiago, Chile, born between 1938 and 1963 (aged 60 to 85 in 2023). Santiago’s 32 metropolitan districts are home to over 40% of Chile’s population. Between July 2023 and January 2024, the survey was carried through a four-stage random sampling design: city block within the 32 districts, dwelling within block, household within dwelling, and finally, an individual aged 60 to 85 within the household. To reduce potential selection bias, the data were weighted with an expansion factor that accounted for selection probability, ineligibility, and unknown eligibility, thereby ensuring representative coverage across districts, age groups, educational levels, and sex. Each participant signed an informed consent form before the interview. The research project was approved by the Ethics Committee for Social Sciences, Humanities, and Arts at the host institution of one of the authors.

To account for the complex multi-stage sampling design, standard errors in all logistic regression models were estimated using the survey weights described above. The expansion factor corrects for differential selection probabilities and non-response across strata. In models estimated without explicit primary sample unit/strata variance correction, this approach provides conservative estimates. Sensitivity analyses with alternative standard error specifications, including design-corrected variants, are available from the authors upon request.

The survey comprises two instruments. The first is a structured questionnaire with 60 items covering employment, family, religion, civic engagement, and political preferences. It includes intergenerational comparisons and a battery of questions on redistributive and tax preferences.

The dependent variables include two measures of policy preferences and redistribution. Interviewees were presented with two options. First, (1) the state should target aid specifically to the poor, or (2) the state should provide assistance to everyone, regardless of whether they are poor or not. This pair, then, maps onto preferences for focalized or universal social benefits. Second, (1) incomes should be made more equal, even if individual effort is not rewarded, or (2) individual effort should be rewarded, even if it leads to significant income differences. This pair captures preferences for income equality vs. merit-based distribution.

Two additional questions assess taxation preferences. Interviewees were presented with two alternatives as well: First, (1) in general, only the richest should pay taxes, or (2) everyone, as citizens, should contribute to the state through taxes according to their means. This pair reflects preferences for mass taxation versus class-based taxation. Second, (1) personal taxes (*impuestos a las personas*) should be the state’s main source of revenue, or (2) the state’s main source of revenue should be taxes on mining and the exploitation of natural resources. This last pair contrasts support for domestic taxation with support for taxes on commodities.

The second survey instrument is a life history calendar designed to reconstruct employment trajectories, capturing pension contributions (as a proxy for formality), occupation histories, and periods of labor market inactivity. Life history calendars help reduce recall bias and situate events chronologically (Belli et al., 2013; Morselli et al., 2013, 2016). Data collection involved face-to-face interviews conducted by trained interviewers in respondents’ homes.

To account for additional sources of variation, we included a set of control variables in our models. Given the established association between left-wing ideology and preferences for universal spending, we control for respondents’ left–right ideological self-placement. In addition, we include controls for educational attainment, gender, beliefs about state efficiency (i.e., whether the state is perceived as making good use of economic resources), and current retirement status (i.e., whether the respondent is retired or active in the workforce). Finally, educational mobility is measured by comparing each respondent’s highest educational attainment to the highest educational level attained by either parent. Table 1 presents descriptive statistics for the dependent variables (rows 1 to 4) and for control variables (rows 5 to 9).

**Table 1:** Descriptive statistics for dependent variables and controls.

Variable	Category	Freq.	Prop.	Mean	SD
Income equality or merit-based distribution	Income equality	305	0.42	—	—
	Merit-based distribution	425	0.58		
Class-based or mass taxation	Class-based	394	0.51	—	—
	Mass taxation	373	0.49	—	—
Focalized or universal social benefits	Focalized benefits	479	0.63	—	—
	Universal social benefits	287	0.37	—	—
Domestic taxation or taxation on natural resources	Domestic taxation	101	0.14	—	—
	Taxation on natural resources	599	0.86	—	—
Gender	Male	242	0.31	—	—
	Female	550	0.69	—	—
Retired	Yes	495	0.63	—	—
	No	295	0.37	—	—
Ideology	Left	121	0.15	—	—
	Center	128	0.16	—	—
	Right	122	0.15	—	—
	Non-ideologue	421	0.53	—	—
Educational mobility	No educational mobility	316	0.40	—	—
	Educational mobility	476	0.60	—	—
Beliefs on state efficiency	Low = 1, 2, 3, 4, High = 5	—	—	2.77	1.19

**Figure 1:**
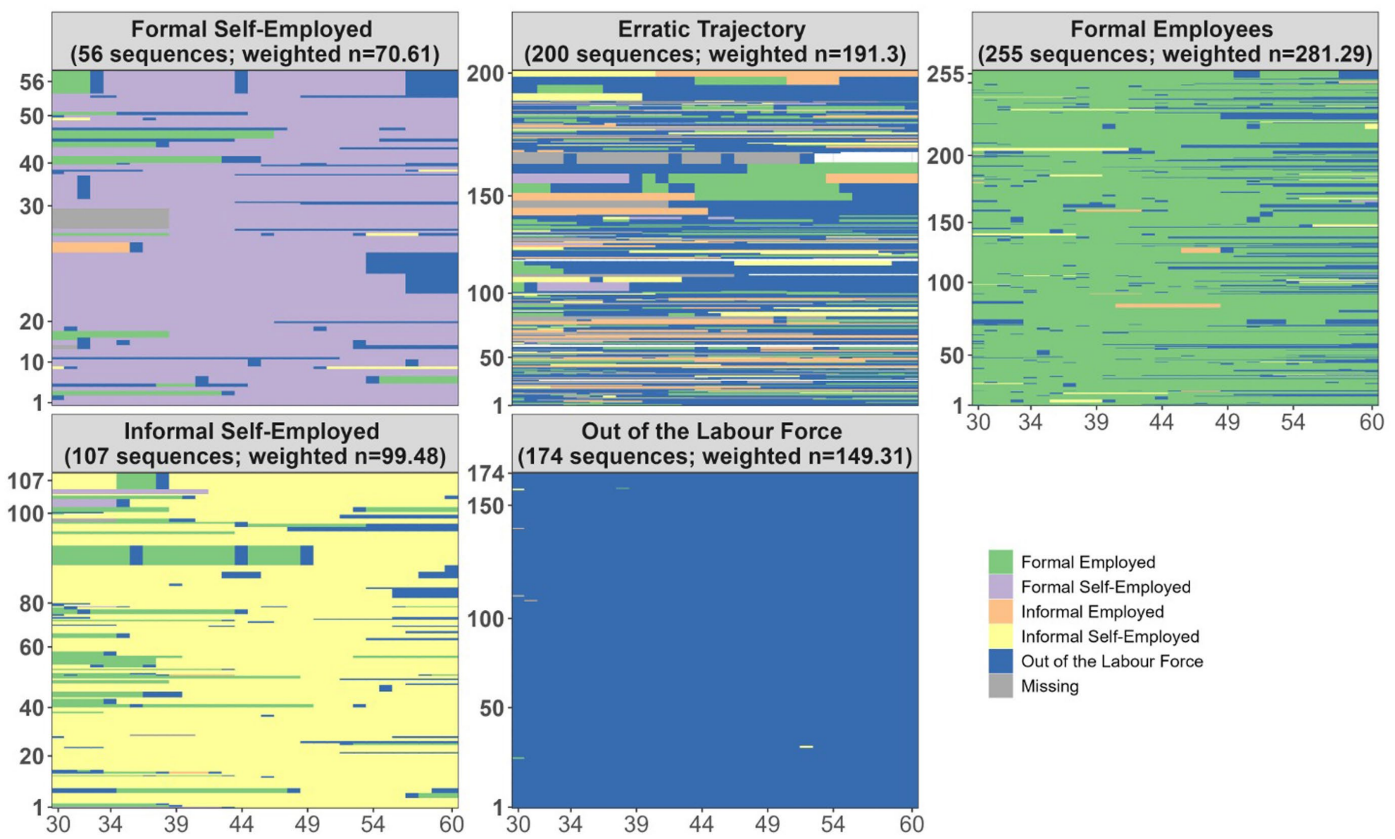
Typologies of labour market trajectories.

Cases with missing values were excluded. The percentage of missing values is as follows: income equality or merit-based distribution (7.83%), class-based or mass taxation (3.16%), focalized or universal social benefits (3.28%), domestic taxation or taxation on natural resources (11.62%), and receiving pension (0.25%). Gender, ideology, and educational mobility had no missing cases. Descriptive statistics of the key explanatory variable are presented in Figure 1. The complete wording of the redistributive preference questions is available in the Appendix.

Several additional sociodemographic variables (age within the cohort, marital status, number of children, and rural–urban origin) were considered but not included in the main models. Given the relatively narrow cohort range and the exclusive focus on metropolitan Santiago, within-sample variation in age and urban origin is limited. Marital status and number of children are excluded because they may partially mediate the relationship between employment trajectories and preferences (e.g., being out of the labor force often reflects family care responsibilities), which could introduce post-treatment bias. We note, however, that including these variables does not substantively alter the main results (available upon request).

The analysis proceeds in two steps. First, the primary independent variables in this study are the employment trajectories reconstructed through sequence analysis (Liao et al., 2022). Second, we used weighted logistic regressions to estimate the effect of these trajectories on redistributive and tax preferences.

## 4 Results

### 4.1 Trajectories

First, we constructed a pseudo-panel of employment trajectories, recording annual statuses from ages 30 to 60 (30 observations per individual), covering a major proportion of working life as well as maximizing the individual observations for the whole trajectory. At each time point, respondents were classified as working or out of the labor force (including unpaid domestic work, unemployment, study, disability, and related categories), self-employed or wage-earner, while pension contribution in each year identified formal vs. informal activities.

We applied sequence analysis (Liao et al., 2022) to compare employment trajectories, using optimal matching to calculate dissimilarities between sequences. The method relies on insertion/deletion and substitution operations, with substitution costs set to two and indel costs to one. This Levenshtein II—inspired scheme emphasizes the order of employment transitions. Pairwise distances were compiled into a dissimilarity matrix and clustered using Ward’s hierarchical agglomerative algorithm (Cabib, 2022; Cornwell, 2015; Liao et al., 2022).

To identify the most reliable cluster solution, we compared fit indices across solutions: Hubert’s Gamma (HG), Point Biserial Correlation (PBC), Average Silhouette Width (ASW), and Hubert’s C (HC). Because these indices operate on different scales (−1 to 1 vs. 0 to 1), we used normalized scores. Higher values of ASW, PBC, and HG, and lower values of HC, indicate better solutions (Studer, 2013). Results of the tests are provided in the Appendix.

Sequence analysis produced five distinct clusters of individuals, each representing a characteristic pattern of labor market engagement across the life course. Incorporating information on part-time versus full-time work generates a solution comparable to the seven trajectories identified by Madero-Cabib et al. (2019). However, given the smaller size of our sample, we retained four clusters based on work/outside the labor market and formal/informal information to ensure sufficient group sizes for analysis. Accordingly, we found complex work histories characterized by erratic patterns that go beyond a simple formal/informal distinction. The trajectories are depicted in Figure 1.

Based on Figure 1, five distinct labor market trajectories can be identified.

The largest group corresponds to formal employees (255 sequences; weighted *n* = 281.29; 35.5%), who maintained stable employment relationships under formal contracts and consistently contributed to the social security system throughout their working lives. The second group includes individuals out of the labor force (174 sequences; weighted *n* = 149.31; 18.9%), who remained predominantly inactive over the observed period, likely due to caregiving responsibilities or other non-paid activities. The third cluster, labeled erratic trajectory (200 sequences; weighted *n* = 191.3; 24.1%), comprises individuals whose employment histories were characterized by frequent transitions between formal, informal, and inactive statuses, reflecting high levels of labor market instability and discontinuity. A fourth group corresponds to the informal self-employed (107 sequences; weighted *n* = 99.48; 12.6%), who engaged in independent activities without formal contracts or social security contributions, showing persistent informality throughout their careers. Finally, the smallest cluster is composed of formal self-employed workers (56 sequences; weighted *n* = 70.61; 8.9%), who operated as independent workers but with formal registration and social security contributions, albeit with some periods of interruption. Overall, labor market trajectories are more fluid than a simple formal/informal dichotomy.

### 4.2 Redistributive preferences

To assess the preferences of individuals who followed these different employment patterns, we calculated four logit models represented by Equation 1:

$$\log(\frac{P(y=1)}{P(y=0)})=\beta_{0}+\beta_{1}{\mathrm{CL}}_{i}+\gamma^{T}X_{i}^{\prime }$$ (1)

where *y* are our dependent variables, namely respondent’s redistributive preferences, measured through four binary outcomes: (a) preferences income equality versus merit-based distribution, (b) preferences for class-based versus mass taxation, (c) preferences for focalized or universal benefits, and (d) preferences for domestic taxation versus taxation on natural resources. In all cases, the variable is coded as 0 for the first option in each pair and 1 for the second. Our key explanatory variable will be the respondents labor trajectory, which will be represented by ${\mathrm{CL}}_{i}$ and its associated effect $\beta_{1}$ . Importantly, to avoid low-*n* bias in our estimates, we recoded the formal self-employed and informal self-employed clusters into a single cluster, namely self-employed (results with the original five cluster solution are in the Appendix). Finally, $X_{i}^{\prime }$ is a vector of individual control variables, and $\gamma^{T}$ is their associated effect. While formal and informal self-employed workers differ in their social security coverage, both groups share the defining characteristic of operating outside dependent wage employment, which may produce similar orientations toward state intervention and taxation despite differences in formal status (Ward, 2019). Full five-cluster results are reported in the Appendix.

Table 2 reports the results of our logistic regression models. Model 1 presents the results for income equality vs. individual effort, Model 2 for taxing the rich vs. general taxes, Model 3 for targeting vs. universal aids, and Model 4 for taxing individuals vs. natural resources. Full model results are available in the Appendix.

**Table 2:** Estimated effects of labor market trajectories on redistribution and taxation preferences.

Variable	Model 1	Model 2	Model 3	Model 4
Intercept	0.422 (0.408)	1.365^***^ (0.403)	−0.259 (0.407)	2.043^***^ (0.595)
Erratic trajectory (ET)	0.145 (0.207)	−0.426^*^ (0.202)	−0.446^*^ (0.208)	−0.100 (0.277)
Out of labor force (OLF)	0.086 (0.222)	−0.544^*^ (0.218)	−0.478^*^ (0.224)	0.206 (0.327)
Self-employed (SE)	−0.091 (0.217)	−0.058 (0.215)	−0.259 (0.215)	0.699^*^ (0.346)
Educational mobility	✓	✓	✓	✓
Gender	✓	✓	✓	✓
Ideology	✓	✓	✓	✓
Retired	✓	✓	✓	✓
Beliefs on state efficiency	✓	✓	✓	✓
AIC	1003.831	1039.370	1017.653	585.038
BIC	1054.324	1090.409	1068.678	635.084
Log likelihood	−490.915	−508.685	−497.827	−281.519
Deviance	981.831	1017.370	995.653	563.038
Num. obs.	728	765	764	699

^***^p < 0.001, ^**^p < 0.01, ^*^p < 0.05.

Coefficients are expressed in logits. All models control for educational mobility, gender, retirement status, and ideological self-positioning, beliefs about state efficiency.

In line with much of the cross-sectional literature, there’s little to separate workers following a variety of trajectories in terms of policy preferences. However, our findings do suggest that relative to those who have been formally employed throughout their working life, individuals with erratic employment histories are significantly less likely to support universal social benefits over targeted ones, with a reduction in the odds of preferring universal benefits of about 36% ( $1-e^{-0.446}=0.36,p<0.05$ ). This contrasts with findings from Europe, where informal workers tend to favor universalism and may reflect the specific configuration of informality in this Latin American setting, where targeted programs often serve as the primary safety net.

In the same vein, the effect of being out of the labor force (OLF) on preferences for targeted versus universal aid is significant at the 95% level. This suggests that individuals who have been predominantly outside the labor force are about 38% less likely to support universal benefits, favoring instead targeted aid $\left(1-e^{-0.478}=0.38,p<0.05\right)$ . A plausible interpretation is that targeted programs may maximize the benefits they themselves could receive, rather than distributing resources more broadly across the entire population.

These results speak directly to H1 and the empirical puzzle identified in the introduction: despite segmented labor markets, redistributive preferences in Latin America do not always align with the expectations derived from studies in advanced economies. The finding that erratic trajectories are associated with support for targeting, rather than universalism, suggests that the instability and unpredictability of employment may foster a pragmatic orientation toward more selective welfare provision, and which places these results closer to research that highlights the opting out by middle classes when benefits are seen as to narrow. In contrast, we find no statistically significant differences across trajectory groups regarding preferences for income equality versus merit-based distribution (H2).

On the other hand, individuals who spent most of their lives outside the labor force are significantly less likely to endorse universal taxation, with a reduction in the odds of about 42% $\left(1-e^{-0.544}=0.42,p<0.05\right)$ , and an increase of the odds of high income taxation of 72% ( $\frac{1}{e^{-0.544}}-1=0.72)$ . This suggests that exclusion from the labor market may shape attitudes toward fiscal justice, making them more prone to endorse class-based taxes compared to those who had more stable labor trajectories (H4). A similar pattern is observed among individuals with an erratic labor trajectory, with a reduction in the odds of roughly 35% $\left(1-e^{-0.426}=0.35,p<0.05\right)$ , that is, a little more than one-third lower likelihood of supporting universal taxation compared to those with stable employment histories, while their odds of supporting high income taxation increases in more than a 50% ( $\frac{1}{e^{-0.426}}-1=0.53$ ).

There are also statistically significant differences in preferences regarding how the state should be funded, through taxes versus natural resources (H5), particularly among the self-employed. According to our results, self-employed individuals are more likely to favor taxation of natural resources, with the odds of doing so being roughly twice as high as those of the reference group $\left(e^{0.699}=2.01,p<0.05\right)$ . To assess whether this effect is driven by individuals with a formal or informal self-employment trajectory, we re-estimated the model using the original trajectories (results are available in Table B3). The estimates indicate that self-employed individuals who contribute to social security (formal self-employed) are more likely to favor taxation of natural resources over domestic taxation (at a 90% confidence level). This category is interesting as it groups self-employed individuals who are treated as formal workers in Chile given their contributions to social security. This effect may stem from the combination of their social security contributions and greater labor uncertainty, which could make this group more resistant to broader forms of taxation than formal employees. Due to the small size of this subgroup, the results are not conclusive; however, they point to potential avenues for further research on H5.

**Figure 2:**
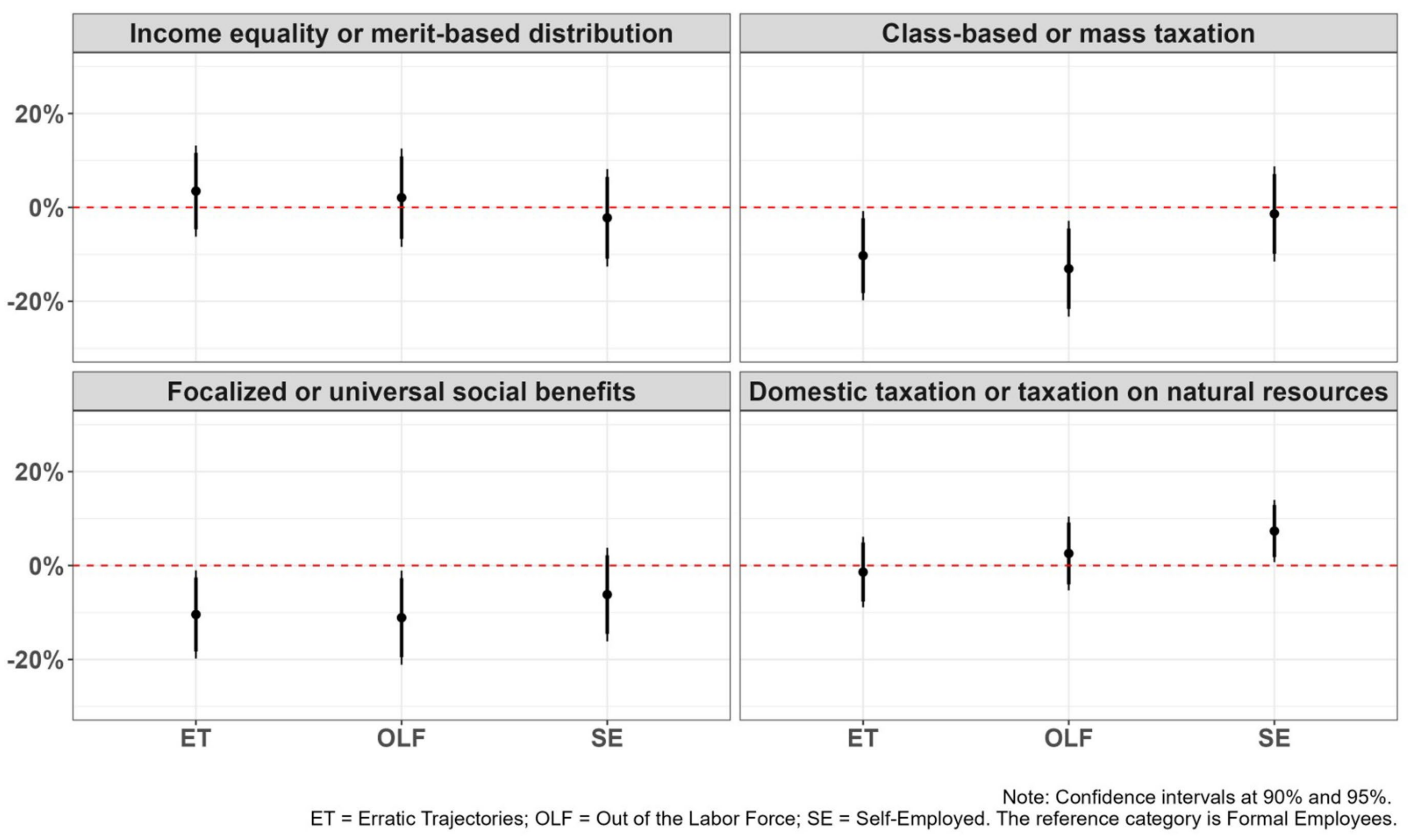
Avarage marginal effects of labor market trajectories on redistribution and taxation preferences.

To better assess the magnitude of the associations, we calculated the average marginal effects for each model (Figure 2). Results indicate that only a few clusters exhibit statistically significant differences at the 95% confidence level. In the class-based versus mass taxation model, belonging to the out of the labor force cluster decreases the predicted probability of supporting class-based taxation by approximately 13 percentage points, compared to the reference group. Similarly, in the focalized versus universal social benefits model, individuals out of the labor force are 11 percentage points less likely to support universal social benefits, suggesting a lower preference for redistributive or inclusive welfare policies among those with weaker labor market attachment. In contrast, in the domestic versus natural resource taxation model, belonging to the self-employed cluster increases the predicted probability of supporting taxation of natural resources by about 7 percentage points, relative to formal employees. No statistically significant effects were observed in the income equality versus merit-based distribution.

All in all, these results highlight how labor market instability, often associated with income volatility, weaker social protection, and more uncertain long-term prospects, also translates into differences in fiscal preferences. Respondents with more vulnerable employment trajectories prefer funding the state through natural resource revenues rather than individual taxation. This logic is consistent: when personal resources are scarce, it may be preferable for the state to mobilize revenues from alternative sources rather than from individuals directly. Alternatively, this means that predominantly formal workers are more likely to develop fiscal citizenship orientations. This result contributes to addressing the second research gap outlined in the introduction, namely, the limited attention paid to taxation preferences in the literature on stratification. It highlights how long-term detachment from formal employment, or even labor insecurity, may foster stronger support for progressive class-based taxation, even in the absence of broader differences in redistributive attitudes. Taken together, these findings indicate that while employment trajectories influence views on the distribution and targeting of social assistance, they may have a more limited effect on broader redistributive preferences.

Moreover, these results remain even after controlling for retirement status. In other words, being retired or receiving a contributory pension has no significant effect on either redistributive or policy preferences. This finding is noteworthy, as much of the existing literature links the receipt of non-contributory benefits to sustained support for more redistributive social policies (Busso et al., 2025; Schwarz and Stampini, 2025). The effect remains even after controlling for ideological self-positioning, a particularly strong predictor for political preferences among Chileans (Bargsted and Somma, 2013; Visconti, 2021).

Finally, the third stage of our analysis examines the role of educational mobility (H3a and H3b). Table 3 reports the results of the interactions between labor market clusters and educational mobility, estimated using the specification in Equation 2:

$$\begin{array}{l} \log(\frac{P(y=1)}{P(y=0)})=\beta_{0}+\beta_{1}{\mathrm{CL}}_{i}+\beta_{2}{\mathrm{EdM}}_{i}\\ +\beta_{3}({\mathrm{CL}}_{i}*{\mathrm{EdM}}_{i})+\gamma^{T}X_{i}^{\prime } \end{array}$$ (2)

where ${\mathrm{EdM}}_{i}$ indicates whether the respondent experienced educational mobility (1 = mobile, 0 = non-mobile), and $\beta_{3}$ captures the moderating effect of educational mobility on the relationship between cluster membership and redistributive preferences. As in Table 1, Model 1 presents the results for income equality vs. individual effort, Model 2 for taxing the rich vs. general taxes, Model 3 for targeting vs. universal aids, and Model 4 for taxing individuals vs. natural resources. Full model results are available in the Appendix.

**Table 3:** Results from generalized models for redistributive preferences with interactions for educational mobility.

Variable	Model 1	Model 2	Model 3	Model 4
Intercept	0.421 (0.424)	1.262^**^ (0.420)	−0.183 (0.425)	1.904^**^ (0.608)
Erratic trajectory (ET)	0.295 (0.329)	0.118 (0.315)	−0.646 (0.333)	0.055 (0.413)
Out of labor force (OLF)	0.062 (0.349)	−0.633 (0.346)	−0.601 (0.352)	0.680 (0.528)
Self-employed (SE)	−0.464 (0.333)	0.015 (0.331)	−0.132 (0.332)	0.896 (0.512)
Upward educational mobility	−0.086 (0.268)	0.116 (0.269)	0.084 (0.267)	0.393 (0.363)
Erratic trajectory (ET)*Educational mobility	−0.264 (0.413)	−0.924^*^ (0.405)	0.329 (0.415)	−0.263 (0.543)
Out of labor force (OLF)*Educational mobility	0.030 (0.429)	0.152 (0.426)	0.205 (0.431)	−0.762 (0.642)
Self-employed (SE)*Educational mobility	0.667 (0.438)	−0.114 (0.433)	−0.229 (0.434)	−0.339 (0.692)
Gender	✓	✓	✓	✓
Ideology	✓	✓	✓	✓
Retired	✓	✓	✓	✓
Beliefs on state efficiency	✓	✓	✓	✓
AIC	1005.584	1037.846	1021.985	589.575
BIC	1069.849	1102.804	1086.925	653.270
Log likelihood	−488.792	−504.923	−496.993	−280.787
Deviance	977.584	1009.846	993.985	561.575
Num. obs.	728	765	764	699

^***^p < 0.001, ^**^p < 0.01, ^*^p < 0.05.

Coefficients are expressed in logits. All models control for gender, retirement status, and ideological self-positioning, beliefs about state efficiency.

Results should be interpreted with caution due to the small sample size. They are best seen as indicative of potential avenues for future research. Indeed, interactions between labor market trajectories and educational mobility yield little as way of results. For example, while long-term labor market inactivity was previously associated with preferences for targeted aid, those who experienced educational mobility despite being outside the labor market tended to favor universal provision. However, since the *p*-values fall well below 90% this would, at best, suggest a hypothesis worth exploring in the future.

In the same vein, results suggest that educational mobility has a modest moderating role over the effect individual’s trajectory and preference for targeted social spending (H3a) and acceptance of inequality (H3b). Positive coefficients are indicative that educational mobility may have reinforced egalitarian orientations among the self-employed but results fall below conventional significance levels.

The most interesting results concern taxation. Educational mobility appears to reduce support for mass domestic taxation among individuals with erratic trajectories (Model 2, *p* < 0.05). Within this group, which comprises a significant share of formally self-employed workers, there seems to be a tendency to favor class-based income taxation, presumably of a progressive kind. In line with broader interpretations of middle-class expansion in Latin America and Chile, these individuals experience some economic opportunity but also face vulnerability and uncertain labor markets (Araujo and Martuccelli, 2014; Dayton-Johnson, 2015; De La O et al., 2025). This pattern may therefore reflect a group that values economic opportunity and even supports private forms of social security (such as individual pension contributions), yet remains resistant to broader, generalized taxation.

## 5 Discussion and limitations

This study examined how long-term employment trajectories shape redistributive and fiscal preferences in a Latin American context. Using sequence analysis, we identified five distinct clusters of labor market engagement, ranging from stable formal employment to persistent exclusion from the labor market.

Complementing quite a substantial body of research who fails to find much to separate formal from informal workers (Baker and Velasco-Guachalla, 2018; Berens, 2015a), our results show, alternatively, that employment instability over time has meaningful implications for attitudes toward social policy design. Compared to consistently formal workers, individuals with erratic trajectories are significantly more likely to support targeted over universal benefits. This finding contrasts with evidence from Europe, where informality often fosters universalist orientations, and suggests that in Latin America targeted programs may be perceived as a reliable safety net. Similarly, individuals outside the labor force also show stronger preferences for targeting, consistent with a logic of maximizing direct benefit receipt when resources are scarce. These results address the first empirical puzzle outlined in the introduction: segmented labor markets in Latin America do not map neatly onto redistributive preferences derived from advanced-economy settings. Instead, instability and exclusion appear to cultivate a pragmatic orientation toward selective provision much in line with historical difficulties in creating cross-class solidarity or more widespread redistribution in Chile and the region (see Maldonado and Canales, 2022; Oxhorn, 1998). Along this last line, we do not find systematic differences across clusters for overall egalitarian versus merit-based orientations (H2).

In the domain of taxation preferences, results are more mixed. Two noteworthy patterns emerge. First, respondents with erratic trajectories and those outside the labor force are more supportive of taxing high incomes, suggesting that labor market exclusion shapes attitudes toward fiscal justice (H4). Second, individuals with vulnerable trajectories are more inclined to favor natural resource revenues over individual taxation, reflecting the expectation that the state should mobilize revenues from sources other than citizens with limited means (H5). These findings can be read in light of recent research that, on the one hand, suggests wide support for progressive taxation of high incomes but which, on the other hand, is more ambivalent toward mass taxation and shared fiscal sacrifice (Atria, 2014; Biehl et al., 2025; Biehl et al., 2019; García-Sánchez et al., 2022).

In this regard, future research could examine how different shades of formality shape economic interests and attitudes toward social policy. The five-cluster employment trajectory solution suggests that individuals already covered by their own contributions but facing greater labor uncertainty, that is, those contributing, technically formal, self-employed workers, tend to hold more ambivalent views on domestic taxation. By contrast, predominantly formal workers appear more inclined to embrace fiscal citizenship, consistent with their sustained engagement in formal contribution systems. Formal employees are not only covered by social security contributions but are also protected in the long run through more stable employment relations and dependent wage-earning positions.

These findings address the second research gap identified in the introduction by highlighting connections between stratification and taxation preferences in Latin America. They suggest that while employment histories may not influence broad redistributive orientations, they do condition attitudes toward how the state should raise and allocate resources.

In addition, these findings carry implications for current fiscal and social policy debates in Chile. The ongoing pension reform and the consolidation of the Pensión Garantizada Universal (PGU) have placed questions of funding (whether through general taxation, payroll contributions, or commodity revenues) at the center of public discourse (Biehl and Montt, 2025). Our results suggest that formally employed workers, who constitute the backbone of the contributory system, are also the segment most likely to endorse broad-based domestic taxation as the appropriate funding mechanism. Conversely, those with erratic or inactive careers, who are also disproportionately dependent on non-contributory transfers, tend to favor natural resource taxation and targeted benefits. This configuration may complicate reform coalitions: while there is broad support for non-contributory expansion, preferences over its funding sources are shaped by long-term labor market experiences in ways that cross-sectional data would not reveal.

The analysis of educational mobility further refines this picture. Having experienced educational mobility, those following erratic trajectories are more supportive of class-taxation. Educational mobility does not affect egalitarian orientations among the formally self-employed but increases support for taxing the wealthy. These heterogeneous effects point to the role of intergenerational resources in moderating the relationship between employment experiences and distributive preferences, though for other dimensions such as targeting versus universalism or taxation sources, the effects of mobility are negligible.

By integrating a life-course perspective into the study of redistributive and fiscal attitudes, this article contributes to understanding how long-term patterns of formality, informality, and exclusion shape individuals’ views. Methodologically, combining retrospective employment data with attitudinal measures bridges life-course sociology and political economy, providing a tool to analyze how the legitimacy of welfare and social spending evolves over time. While policy preferences may themselves change, examining the dynamics of employment trajectories allows us to capture the processual determinants of current preferences. This offers insights that can also inform comparative debates on welfare attitudes.

Future research could extend the analysis beyond life-course trajectories to include generational comparisons. This study examined a cohort that experienced several institutional configurations of social security, most recently a decisive shift toward non-contributory pension and health benefits. Younger generations, who face a markedly different labor market and have been socialized within a distinct institutional context, may therefore hold and develop attitudes toward social policy that diverge significantly from those of older cohorts. In addition, while the present study controls for gender, it does not explore how the relationship between trajectories and preferences may differ for men and women, whose labor market participation patterns (particularly in the out of the labor force cluster, likely dominated by women in caregiving roles) may carry distinct normative meanings and policy orientations (Cabib et al., 2026b).

This article frames its contribution as identifying associations between employment trajectories and policy preferences rather than estimating causal effects. Ideology, institutional trust, and state efficiency perceptions are treated as control variables in our models, though they may partially mediate the relationship between trajectories and preferences. The patterns documented here are best understood as evidence of welfare attitudes rather than as causal claims, which we will develop in future research.

While our results align with existing research on taxation preferences and the limits of solidarity in redistributive attitudes, the study has several limitations. First, the reliance on retrospective survey data raises the possibility of recall bias and measurement error in reported employment histories. This form of measurement error may be non-random, stratified, and could attenuate estimated differences between trajectory groups.

Second, the sample is limited to a single cohort, which constrains the generalizability of the findings to other age groups or younger generations whose labor market experiences have unfolded under different institutional settings. Beyond age-group generalizability, the focus on metropolitan Santiago, which is more formally employed than rural areas or smaller cities, may limit extrapolation to other Chilean contexts or to other Latin American countries with lower rates of formalization, different welfare policies, or distinct political histories. While Chile’s experience under privatized pensions and expanding non-contributory benefits represents a theoretically relevant case for examining the preferences of older workers, caution is warranted in applying these findings to younger cohorts or to other countries.

Third, several survey items measure attitudes in broad terms, limiting our ability to capture more nuanced preferences toward specific policies or programs with which respondents may identify or be more familiar. Future research using conjoint experimental measures would allow finer-grained assessment of these preference structures. The assignment of individuals to trajectory clusters involves statistical uncertainty that is not propagated into the regression stage; treating cluster membership as fixed may lead to somewhat underestimated standard errors. The Appendix reports cluster validity indices (ASW, HG, PBC, HGSD, and HC) across solutions from 2 to 14 clusters, confirming the five-cluster solution as the most consistent and statistically robust configuration (Figures A2–A4). Finally, the relatively small sample size required a parsimonious clustering solution, which may have obscured finer-grained variation in employment trajectories.

Additional limitations concern the study design and measurement. First, the individual-level analytical framework does not capture household-level dynamics: a respondent’s preferences may be shaped by the employment trajectories of co-residing family members (e.g., a formally employed spouse or self-employed child). Second, the operationalization of formality through pension contributions, while tractable and consistent with established approaches in the literature (Madero-Cabib et al., 2019), does not capture the full multidimensionality of employment quality: workers can be formally registered yet in precarious conditions, or informally employed yet economically stable. Third, the study is subject to survivor bias inherent in retrospective cohort analysis: individuals with the most precarious and vulnerable labor histories may have faced higher mortality rates and are therefore underrepresented, potentially attenuating associations between vulnerable trajectories and preferences.

Overall, our findings suggest that while employment patterns are more fluid among individuals whose trajectories alternate between stable formality and extended inactivity, these experiences do not necessarily generate shared belief systems, as much of the cross-sectional literature documents. In fact, the more formal stable clusters display conventional preferences for mass taxation, whereas those with erratic trajectories or prolonged detachment from the labor force continue to legitimize targeted spending and class-based taxation. Despite the prevalence of informality within households and individual biographies, segmented work paths, in other words, do not necessary lead to shared beliefs.

## Data Availability

The raw data supporting the conclusions of this article will be made available by the authors, without undue reservation.
