# Supplementary material for: Segmented paths, shared beliefs? Employment histories and welfare preferences in Chile
Source: Front Sociol. 2026 Apr 20;11:1735350. doi: 10.3389/fsoc.2026.1735350 (PMC13135952; doi:10.3389/fsoc.2026.1735350)
Supplement: Supplementary file 1 [file Data_Sheet_1.pdf]

Segmented paths, shared beliefs? Employment histories and welfare preferences in Chile - Online Appendix

1 Dependent Variable

Our independent variables are captured with four different variables:

1. **Income equality or merit-based distribution**
   Survey item: Income should be made more equal, even if individual effort is not rewarded **vs.** Individual effort should be rewarded even if this results in large income differences.
   Measurement: This variable captures respondents' preference for **redistributive equality** versus **rewarding individual effort**.
   - 0 = Support for income equality (favor redistribution)
   - 1 = Support for merit-based income (favor individual effort)
2. **Class-based or mass taxation**
   Survey item: In general, only the richest should pay taxes **vs.** All citizens should contribute taxes to the state according to their means.
   Measurement: This variable captures attitudes toward whether **only the rich** should bear the tax burden (**class-based taxation**) or **everyone** should contribute according to their means (**mass taxation**).
   - 0 = Only the rich should pay taxes (class-based taxation)
   - 1 = Everyone should pay according to their means (mass taxation)
3. **Focalized or universal social benefits**
   Survey item: In general, the state should focus its assistance on the poorest **vs.** In general, the state should help everyone regardless of whether they are poor or not.
   Measurement: This variable captures preference for **targeted welfare policies** focused on the poor versus **universal welfare policies** available to all.
   - 0 = Focalized benefits (aid targeted at the poor)
   - 1 = Universal benefits (aid for everyone)
4. **Domestic taxation or taxation on natural resources**
   Survey item: Taxes on individuals should be the main source of state revenue **vs.** The main source of state revenue should be taxes on mining and natural resource exploitation.

Measurement: This variable captures the respondent's preference for financing the state through **domestic personal taxation** or through **natural resource taxation**.

- 0 = Domestic taxation (mainly from individuals)
- 1 = Natural resource taxation (mainly from mining and resource extraction)

2 Independent Variable and Control Variables

2.1 Independent variable: labour trajectories

Our key independent variable is respondent's labor trajectory. We grouped the respondents into five clusters, each representing a different labor trajectory. To build these clusters, we relied on a life calendar survey.

We asked respondents to list up to ten jobs they have had throughout their lives, indicating the occupation and place of work for each job. Then, they marked the specific periods of time during which they worked in each job using a horizontal line across the corresponding years (as illustrated in the calendar).

Afterward, for each job (from Job 1 to Job 10), respondents were asked to specify additional characteristics of their employment, including whether they contributed to a pension during most of the time and what type of work they performed, as a proxy of formality. In particular, they were asked whether, in that job, they were:

- Employee
- Self-employed on a fee basis
- Self-employed on their own account
- Employer or business owner

We run a sequence analysis using the TraMineR R package (version 2.2-11). Results of the sequence are depicted in Figure A1. The figure displays the individual labor trajectories of respondents from age 30 to 60. Each horizontal line represents one respondent's employment sequence over time (weighted N = 792). Colors indicate different labor-market states: formal employed, informal employed, formal self-employed, informal self-employed, out of the labor force, and missing information.

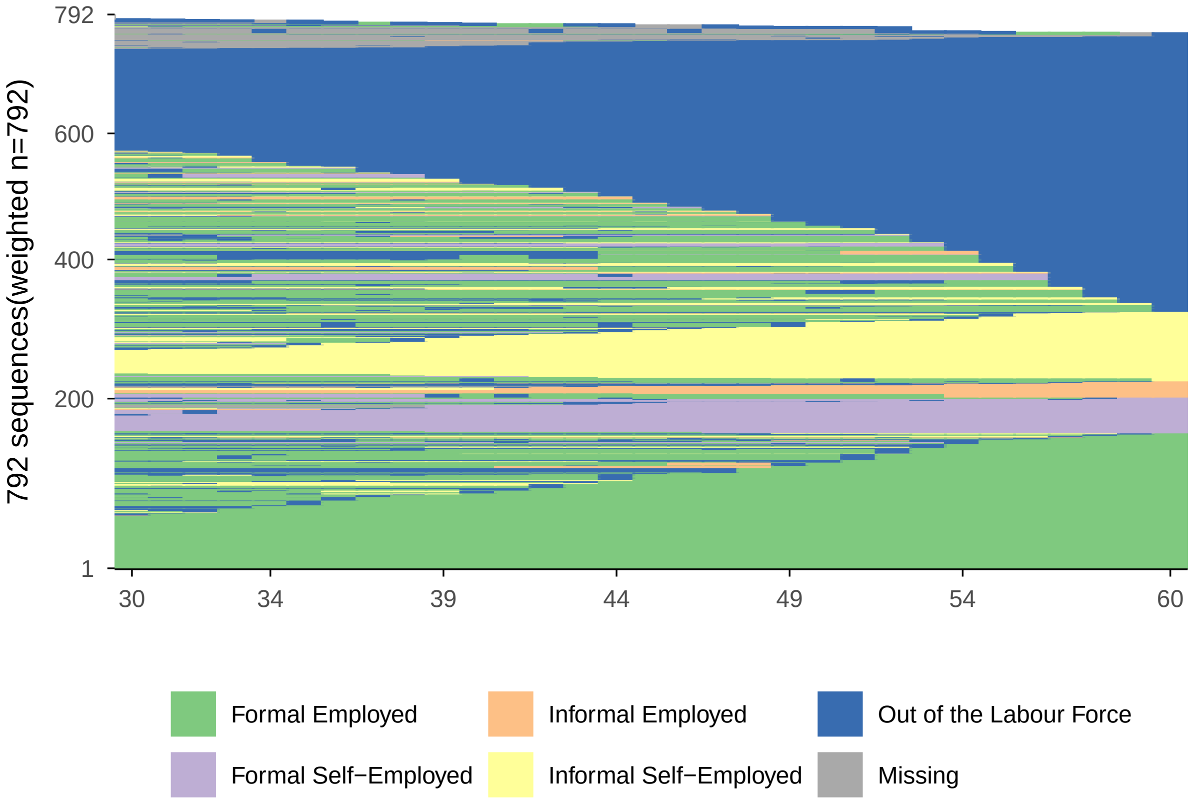

Figure A 1: Employment trajectories over the life course

As can be observed, a significant share of respondents spent large portions of their working lives in formal employment (green) or out of the labor force (blue), while others experienced frequent transitions between informal employment and self-employment (yellow and orange). The upper part of the plot reveals an accumulation of time spent out of the labor force toward later ages, consistent with retirement patterns.

Figures 2 to 4 report the results of the cluster validity analyses, showing that the five-cluster solution achieves the highest average silhouette width and consistent performance across multiple validity indices (ASW, HG, PBC, HGSD, and HC), supporting its selection as the optimal number of clusters used in our *estimates*.

The Average Silhouette Width (ASW) evaluates the compactness and separation of clusters, where higher values indicate a better-defined structure. Hubert's Gamma (HG) measures the correlation between the dissimilarity matrix and the partition structure, while the Point-Biserial Correlation (PBC) similarly assesses the degree of separation among clusters. The Hubert's Gamma Standardized Difference (HGSD) provides a normalized version that allows better comparability across solutions. The C-index (HC) quantifies the ratio of within-cluster to between-cluster distances, with lower values indicating more compact and distinct clusters.

By comparing all these indices, the five-cluster solution emerges as the most consistent and statistically robust configuration, balancing within-cluster homogeneity and between-cluster separation.

This evidence justifies its use in the subsequent estimations.

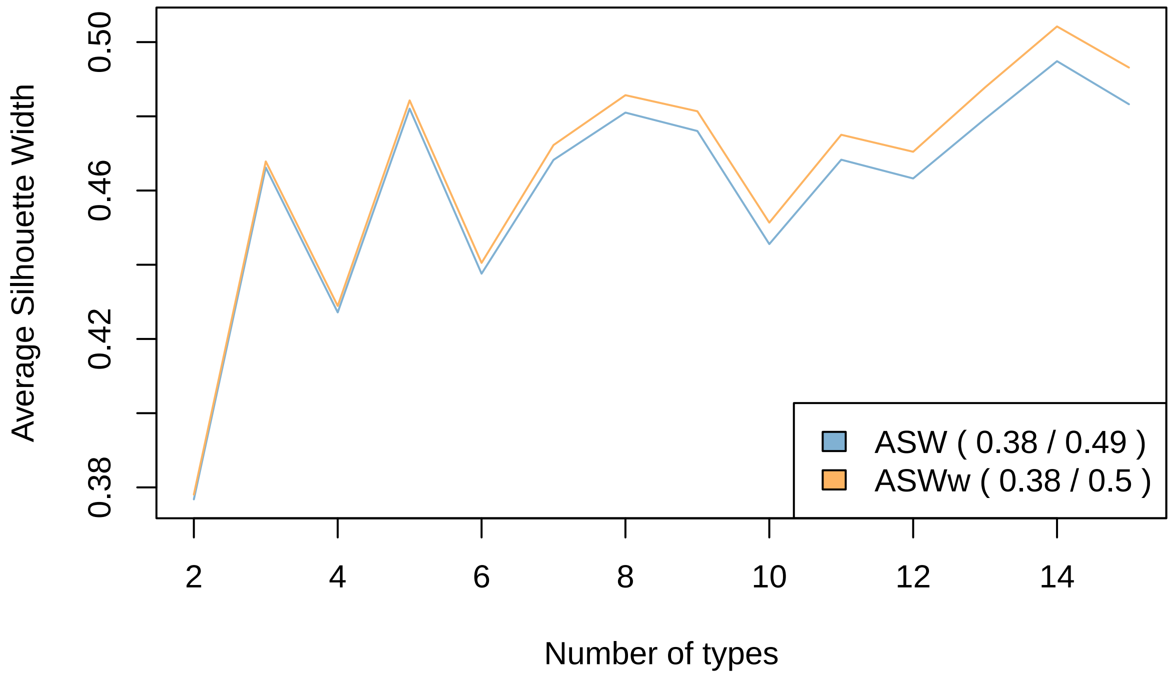

Figure A 2: Cluster quality measures based on Average Silhouette Width (ASW)

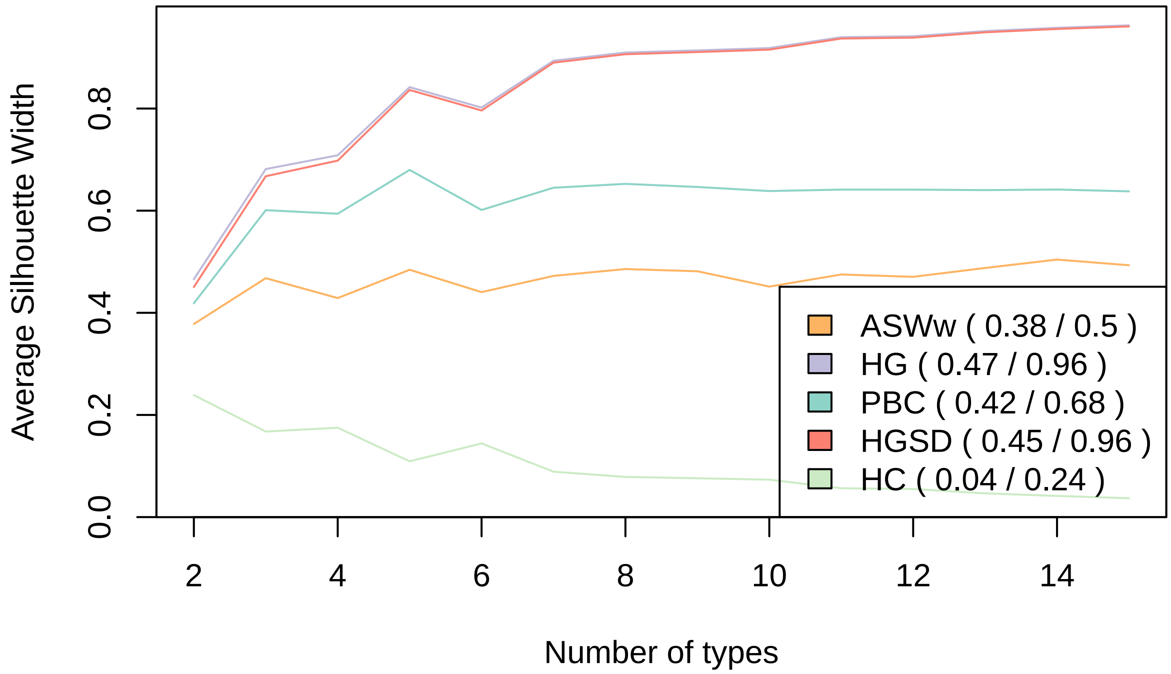

Figure A 3: Comparison of clustering validity indices (ASW, HG, PBC, HGSD, and HC)

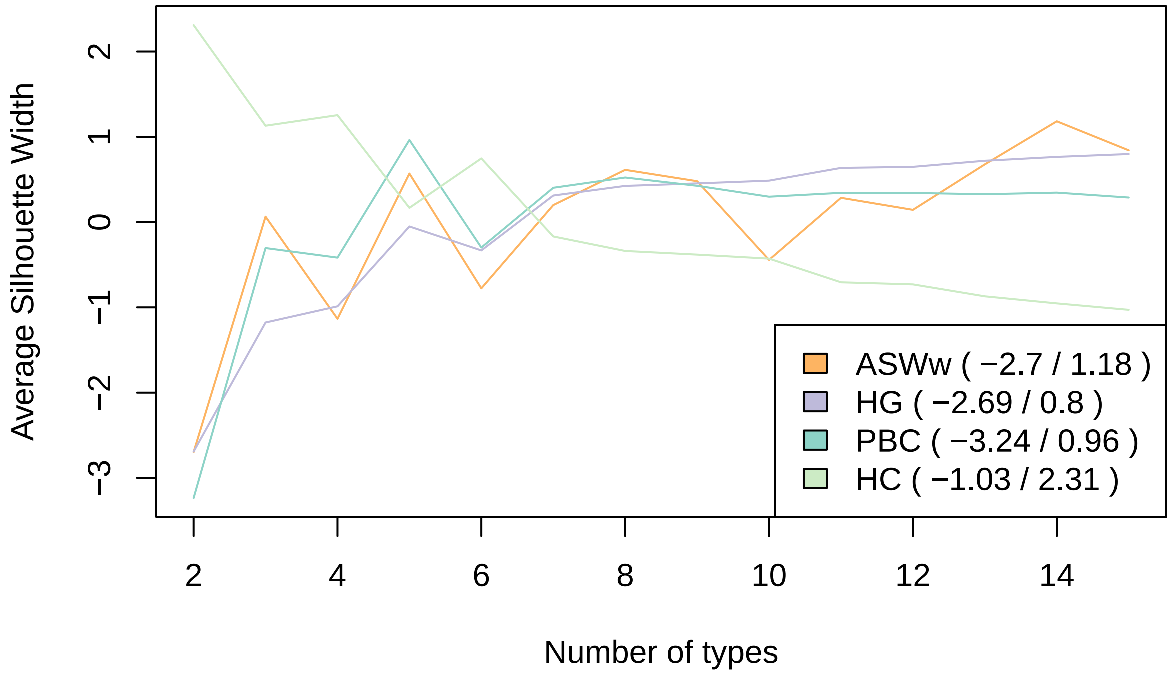

Figure A 4: Standardized clustering validity indices (z-score normalization)

2.2 Control variables

We included a set of control variables, namely:

- Ideological self-positioning: we asked respondents for locate themselves in an ideological scale, using the following question: *Traditionally, in our country people define political positions as being closer to the left, the center, or the right. On a scale from 0 to 10, where 0 represents being on the "left," 5 represents being at the "center," and 10 represents being on the "right," the numbers between 1 and 4 represent nuances between being "left" and "center-left," while the numbers between 6 and 9 represent nuances between being "center-right" and "right." Where would you place yourself on this scale today? Please indicate the number that best fits you.*. We grouped individuals that responded 0 to 4 as (1) Left, 5 as (2) Center, 6 to 10 as (3) Right, and those who did not responded as (4) Non-ideologues.

- Beliefs on State Efficiency: This variable was constructed based on the following survey question: *Taxes are used to help those most in need.* Respondents were asked to indicate their level of agreement with this statement on a five-point Likert scale ranging from 1 (Strongly disagree), 2 (Disagree), 3 (Neither agree nor disagree), 4 (Agree), to 5 (Strongly agree).

- Retirement status: we asked respondents *Are you already retired.* 1 = Retired, 2 = Non-retired

- **Educational mobility:** We constructed the indicator using the respondent's attained educational level, which was captured with the following question: *Please look at the following card. What is the highest educational level you have completed? 1. Never attended school or attended only a few years of primary school; 2. Primary education (elementary or preparatory); 3. Secondary education (humanities or general studies); 4. Technical, commercial, industrial, or teacher training school; 5. Incomplete technical education (1–3 year programs); 6. Complete technical education (1–3 year programs); 7. Incomplete professional education (programs of 4 years or more); 8. Complete professional education (programs of 4 years or more); 9. Incomplete postgraduate education (master's or doctoral); 10. Complete postgraduate education (master's or doctoral).*

  Respondents were then asked to report the highest educational level attained by their father and mother, respectively, with the following question: *What was the highest educational level attained by your father/mother?*. Individuals who attained a higher educational level than either or both of their parents were coded as 1, whereas those who attained an equal or lower level were coded as 0.

- Gender: we asked respondents gender, where 0 = Men and 1 = Women.

3 Complete Models

Tables B1 and B2 report the complete results of the baseline models estimated using standard logistic regression.

	Model 1	Model 2	Model 3	Model 4
Intercept	0.422	1.365^{***}	−0.259	2.043^{***}
	(0.408)	(0.403)	(0.407)	(0.595)
Erratic Trajectory (ET)	0.145	-0.426^{*}	-0.446^{*}	−0.100
	(0.207)	(0.202)	(0.208)	(0.277)
Out of Labor Force (OLF)	0.086	-0.544^{*}	-0.478^{*}	0.206
	(0.222)	(0.218)	(0.224)	(0.327)
Self-Employed (SE)	−0.091	−0.058	−0.259	0.699^{*}
	(0.217)	(0.215)	(0.215)	(0.346)
Upward Educational Mobility	−0.009	−0.115	0.160	0.124
	(0.155)	(0.152)	(0.155)	(0.221)
Women	−0.110	-0.566^{**}	−0.109	0.200
	(0.177)	(0.174)	(0.177)	(0.249)
Non-Retired	0.066	−0.004	0.319^{*}	0.135
	(0.162)	(0.159)	(0.161)	(0.239)
Beliefs on State Efficiency	−0.063	-0.143^{*}	−0.013	$-0.174^{\cdot}$
	(0.064)	(0.063)	(0.064)	(0.092)
Center	0.091	0.558^{*}	−0.043	−0.062
	(0.264)	(0.266)	(0.270)	(0.428)
Right	0.539^{*}	$0.452^{\cdot}$	0.272	−0.423
	(0.273)	(0.268)	(0.270)	(0.403)
Non-ideologue	0.239	0.152	−0.040	−0.514
	(0.219)	(0.219)	(0.221)	(0.343)
AIC	1003.831	1039.370	1017.653	585.038
BIC	1054.324	1090.409	1068.678	635.084
Log Likelihood	−490.915	−508.685	−497.827	−281.519
Deviance	981.831	1017.370	995.653	563.038
Num. obs.	728	765	764	699

$^{***}p < 0.001$; $^{**}p < 0.01$; $^{*}p < 0.05$; $^{\cdot}p < 0.1$

Table B 1: Full logit models for redistributive preferences with four cluster solutions

	Model 1	Model 2	Model 3	Model 4
Intercept	0.421	1.262^{**}	−0.183	1.904^{**}
	(0.424)	(0.420)	(0.425)	(0.608)
Erratic Trajectory (ET)	0.295	0.118	$-0.646^{\cdot}$	0.055
	(0.329)	(0.315)	(0.333)	(0.413)
Out of Labor Force (OLF)	0.062	$-0.633^{\cdot}$	$-0.601^{\cdot}$	0.680
	(0.349)	(0.346)	(0.352)	(0.528)
Self-Employed (SE)	−0.464	0.015	−0.132	$0.896^{\cdot}$
	(0.333)	(0.331)	(0.332)	(0.512)
Upward Educational Mobility	−0.086	0.116	0.084	0.393
	(0.268)	(0.269)	(0.267)	(0.363)
Women	−0.094	-0.591^{***}	−0.121	0.200
	(0.179)	(0.175)	(0.177)	(0.250)
Non-Retired	0.065	0.018	0.319^{*}	0.126
	(0.163)	(0.160)	(0.162)	(0.239)
Beliefs on State Efficiency	−0.056	-0.140^{*}	−0.017	$-0.177^{\cdot}$
	(0.064)	(0.064)	(0.064)	(0.093)
Center	0.097	0.550^{*}	−0.042	−0.066
	(0.265)	(0.267)	(0.270)	(0.428)
Right	0.546^{*}	$0.446^{\cdot}$	0.274	−0.436
	(0.274)	(0.269)	(0.270)	(0.403)
Non-ideologue	0.243	0.125	−0.037	−0.523
	(0.220)	(0.220)	(0.222)	(0.344)
Erratic Trajectory (ET)*Educational Mobility	−0.264	-0.924^{*}	0.329	−0.263
	(0.413)	(0.405)	(0.415)	(0.543)
Out of Labor Force (OLF)*Educational Mobility	0.030	0.152	0.205	−0.762
	(0.429)	(0.426)	(0.431)	(0.642)
Self-Employed (SE)*Educational Mobility	0.667	−0.114	−0.229	−0.339
	(0.438)	(0.433)	(0.434)	(0.692)
AIC	1005.584	1037.846	1021.985	589.575
BIC	1069.849	1102.804	1086.925	653.270
Log Likelihood	−488.792	−504.923	−496.993	−280.787
Deviance	977.584	1009.846	993.985	561.575
Num. obs.	728	765	764	699

$^{***}p < 0.001$; $^{**}p < 0.01$; $^{*}p < 0.05$; $^{\cdot}p < 0.1$

Table B 2: Full logit models for redistributive preferences with four cluster solutions (with interactions)

Table B3 presents the corresponding models estimated using survey-weighted logistic regression, accounting for the complex sampling design. Specifically, we incorporate expansion weights and primary sampling units (PSU), clustering observations at the level of the sampling unit, and compute standard errors using Taylor linearization. This approach corrects for unequal selection probabilities and intra-cluster correlation, yielding design-consistent estimates. As expected, coefficient estimates remain substantively similar, while standard errors are generally larger compared to the unweighted models.

	Model 1	Model 2	Model 3	Model 4
Intercept	0.675	1.515^{**}	-0.057	1.728^{*}
	(0.446)	(0.565)	(0.534)	(0.717)
Erratic Trajectory (ET)	0.135	-0.626^{*}	-0.555^{*}	0.335
	(0.201)	(0.247)	(0.239)	(0.342)
Out of Labor Force (OLF)	0.188	-0.389	$-0.545^{\cdot}$	0.389
	(0.267)	(0.406)	(0.308)	(0.440)
Self-Employed (SE)	0.221	-0.081	-0.273	-0.020
	(0.339)	(0.263)	(0.271)	(0.362)
Women	-0.025	-0.493^{*}	-0.076	-0.033
	(0.215)	(0.212)	(0.204)	(0.288)
Not Retired	0.033	0.137	0.454^{*}	-0.130
	(0.214)	(0.250)	(0.189)	(0.304)
Upward Educational Mobility	-0.137	-0.154	-0.237	0.168
	(0.230)	(0.162)	(0.197)	(0.280)
Good Use of Resources by the Government	-0.118^{*}	-0.301^{**}	-0.045	-0.060
	(0.059)	(0.091)	(0.116)	(0.152)
Center	-0.270	$0.609^{\cdot}$	-0.158	0.600
	(0.336)	(0.330)	(0.354)	(0.517)
Right	0.436	0.813^{**}	0.201	0.188
	(0.357)	(0.303)	(0.323)	(0.478)
Non-ideologue	-0.073	0.321	0.085	0.225
	(0.263)	(0.244)	(0.265)	(0.439)
Deviance	974.692	995.040	991.489	522.224
Dispersion	1.000	1.002	1.006	0.995
Num. obs.	728	765	764	699

$^{***}p < 0.001$; $^{**}p < 0.01$; $^{*}p < 0.05$; $^{\cdot}p < 0.1$

Table B 3: Weighted logit models for redistributive preferences with four cluster solutions

3.1 Five clusters complete models

Tables B4 and B5 report the results for the non-recodified cluster solution.

	Model 1	Model 2	Model 3	Model 4
Intercept	0.413	1.376***	−0.259	2.020***
	(0.409)	(0.405)	(0.408)	(0.596)
Erratic Trajectory (ET)	0.143	−0.425*	−0.446*	−0.105
	(0.207)	(0.202)	(0.208)	(0.277)
Out of Labor Force (OLF)	0.083	−0.541*	−0.478*	0.200
	(0.223)	(0.219)	(0.225)	(0.327)
Informal Self-Employed (ISE)	−0.131	−0.010	−0.259	0.564
	(0.248)	(0.248)	(0.247)	(0.386)
Formal Self-Employed (FSE)	−0.009	−0.146	−0.259	1.043$^{\cdot}$
	(0.330)	(0.315)	(0.324)	(0.628)
Upward Educational Mobility	−0.011	−0.112	0.160	0.116
	(0.155)	(0.153)	(0.155)	(0.222)
Women	−0.105	−0.571**	−0.109	0.212
	(0.178)	(0.174)	(0.177)	(0.249)
Non-Retired	0.069	−0.007	0.319*	0.138
	(0.162)	(0.160)	(0.162)	(0.239)
Beliefs on State Efficiency	−0.062	−0.145*	−0.013	−0.169$^{\cdot}$
	(0.064)	(0.064)	(0.064)	(0.093)
Center	0.091	0.560*	−0.043	−0.066
	(0.264)	(0.266)	(0.270)	(0.428)
Right	0.535$^{\cdot}$	0.457$^{\cdot}$	0.272	−0.436
	(0.274)	(0.268)	(0.270)	(0.403)
Non-ideologue	0.239	0.153	−0.040	−0.513
	(0.219)	(0.219)	(0.221)	(0.343)
AIC	1005.721	1041.224	1019.653	586.524
BIC	1060.805	1096.902	1075.316	641.120
Log Likelihood	−490.861	−508.612	−497.827	−281.262
Deviance	981.721	1017.224	995.653	562.524
Num. obs.	728	765	764	699

$^{***}p < 0.001$; $^{**}p < 0.01$; $^{*}p < 0.05$; $^{\cdot}p < 0.1$

Table B 4: Full logit models for redistributive preferences with five cluster solutions

	Model 1	Model 2	Model 3	Model 4
Intercept	0.450	1.323**	−0.203	1.877**
	(0.427)	(0.424)	(0.426)	(0.610)
Erratic Trajectory (ET)	0.300	0.130	−0.649$^{\cdot}$	0.051
	(0.330)	(0.316)	(0.333)	(0.413)
Out of Labor Force (OLF)	0.066	−0.622$^{\cdot}$	−0.606$^{\cdot}$	0.673
	(0.349)	(0.346)	(0.352)	(0.528)
Informal Self-Employed (ISE)	−0.355	0.230	−0.238	0.782
	(0.368)	(0.369)	(0.369)	(0.552)
Formal Self-Employed (FSE)	−0.760	−0.543	0.153	1.315
	(0.542)	(0.530)	(0.533)	(1.073)
Upward Educational Mobility	−0.084	0.119	0.083	0.392
	(0.268)	(0.270)	(0.267)	(0.363)
Women	−0.102	−0.610***	−0.117	0.211
	(0.180)	(0.177)	(0.178)	(0.251)
Non-Retired	0.068	0.017	0.321*	0.130
	(0.163)	(0.161)	(0.162)	(0.239)
Beliefs on State Efficiency	−0.057	−0.143*	−0.017	−0.172$^{\cdot}$
	(0.065)	(0.064)	(0.064)	(0.093)
Center	0.084	0.529*	−0.030	−0.070
	(0.266)	(0.268)	(0.271)	(0.429)
Right	0.530$^{\cdot}$	0.426	0.287	−0.449
	(0.275)	(0.270)	(0.272)	(0.405)
Non-ideologue	0.221	0.089	−0.019	−0.522
	(0.221)	(0.221)	(0.223)	(0.345)
Erratic Trajectory (ET)*Educational Mobility	−0.268	−0.931*	0.332	−0.262
	(0.413)	(0.405)	(0.415)	(0.543)
Out of Labor Force (OLF)*Educational Mobility	0.029	0.151	0.205	−0.761
	(0.429)	(0.427)	(0.431)	(0.641)
Informal Self-Employed (ISE)*Educational Mobility	0.416	−0.429	−0.050	−0.385
	(0.497)	(0.497)	(0.493)	(0.769)
Formal Self-Employed (FSE)*Educational Mobility	1.220$^{\cdot}$	0.590	−0.636	−0.443
	(0.695)	(0.662)	(0.673)	(1.321)
AIC	1008.445	1039.733	1025.356	593.030
BIC	1081.889	1113.971	1099.574	665.825
Log Likelihood	−488.222	−503.866	−496.678	−280.515
Deviance	976.445	1007.733	993.356	561.030
Num. obs.	728	765	764	699

$^{***}p < 0.001$; $^{**}p < 0.01$; $^{*}p < 0.05$; $^{\cdot}p < 0.1$

Table B 5: Full logit models for redistributive preferences with five cluster solutions (with interactions)

4 Interaction by gender

Table B6 reports the results of the models with interactions between the cluster variable and the respondent's gender.

	Model 1	Model 2	Model 3	Model 4
Intercept	-0.210	1.045^{*}	-0.103	2.292^{**}
	(0.504)	(0.510)	(0.503)	(0.722)
Erratic Trajectory (ET)	0.721	0.514	-1.050	-0.015
	(0.876)	(0.841)	(0.887)	(1.116)
Out of Labor Force (OLF)	2.029	-0.703	-1.530	-1.616
	(1.254)	(1.094)	(1.255)	(1.362)
Self-Employed (SE)	$1.254^{\cdot}$	0.568	-0.140	0.476
	(0.701)	(0.696)	(0.681)	(1.084)
Upward Educational Mobility	-0.021	-0.135	0.151	0.111
	(0.156)	(0.153)	(0.156)	(0.222)
Women	0.295	-0.354	-0.202	0.061
	(0.263)	(0.265)	(0.262)	(0.364)
Non-Retired	0.120	0.013	$0.290^{\cdot}$	0.090
	(0.165)	(0.162)	(0.164)	(0.244)
Beliefs on State Efficiency	-0.056	-0.142^{*}	-0.013	$-0.182^{\cdot}$
	(0.065)	(0.063)	(0.064)	(0.093)
Center	0.065	0.555^{*}	-0.040	-0.045
	(0.266)	(0.266)	(0.270)	(0.429)
Right	$0.487^{\cdot}$	0.438	0.280	-0.390
	(0.275)	(0.269)	(0.271)	(0.405)
Non-ideologue	0.224	0.139	-0.042	-0.515
	(0.220)	(0.219)	(0.222)	(0.344)
Erratic Trajectory*Women	-0.382	-0.547	0.347	-0.024
	(0.488)	(0.471)	(0.493)	(0.629)
Out of Labor Force*Women	$-1.104^{\cdot}$	0.046	0.574	1.017
	(0.667)	(0.590)	(0.667)	(0.761)
Self-Employed*Women	-0.883^{*}	-0.406	-0.078	0.150
	(0.435)	(0.430)	(0.429)	(0.693)
AIC	1004.169	1043.399	1022.247	589.183
BIC	1068.433	1108.357	1087.187	652.878
Log Likelihood	-488.084	-507.700	-497.124	-280.591
Deviance	976.169	1015.399	994.247	561.183
Num. obs.	728	765	764	699

$^{***}p < 0.001$; $^{**}p < 0.01$; $^{*}p < 0.05$; $^{\cdot}p < 0.1$

Table B 6: Full logit models for redistributive preferences with four cluster solutions
